# Updated Progress of the Copper-Catalyzed Borylative Functionalization of Unsaturated Molecules

**DOI:** 10.3390/molecules28052252

**Published:** 2023-02-28

**Authors:** Bingru Li, Huayu Liang, Arumugam Vignesh, Xiaoyu Zhou, Yan Liu, Zhuofeng Ke

**Affiliations:** 1 School of Materials Science and Engineering, PCFM Lab, Sun Yat-sen University, Guangzhou 510275, China; 2 School of Chemical Engineering and Light Industry, Guangdong University of Technology, Guangzhou 510006, China; 3 Guangdong Provincial Key Laboratory of Plant Resources Biorefinery, Guangzhou 510275, China

**Keywords:** copper, borylation, multiple bonds, hydroboration, carboborylation

## Abstract

Borylation has become a powerful method to synthesize organoboranes as versatile building blocks in organic synthesis, medicinal chemistry, and materials science. Copper-promoted borylation reactions are extremely attractive due to the low cost and non-toxicity of the copper catalyst, mild reaction conditions, good functional group tolerance, and convenience in chiral induction. In this review, we mainly updated recent advances (from 2020 to 2022) in the synthetic transformations in C=C/C≡C multiple bonds, and C=E multiple bonds mediated by copper boryl systems.

## 1. Introduction

**Figure 1:**
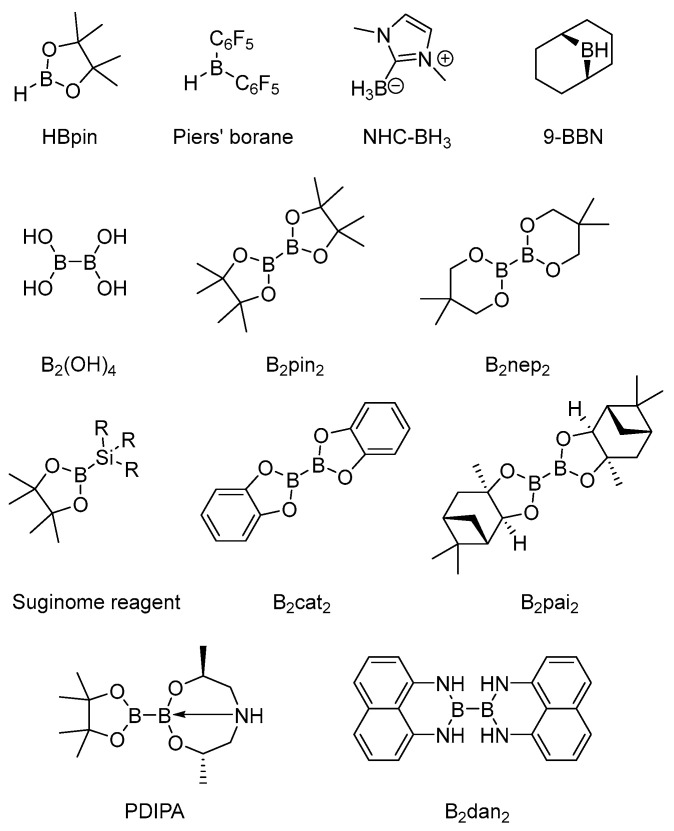
Commonly used boron reagents involved in Cu-boryl promoted reactions.

Organoboron-containing molecules have diverse reactivities and versatile properties in organic chemistry, medicinal chemistry, and materials science [1]. The formed boron-containing molecules can be converted into different functional groups such as hydroxy groups, amino groups, halogens, etc., and especially the boryl compounds are important cross-coupling partners in organic synthesis. Thus, organoboron-containing molecules have been attracting numerous effects towards their synthesis [2]. Transition metal catalysis has led to a variety of synthetic strategies and methods for the synthesis of organoboron compounds [3,4,5,6]. Copper-boryl-promoted borylation reactions are some of the most powerful for transition metal catalysis [5,7, which have several advantages including: (1) using an abundant and environmentally benign metal; (2) mild reaction conditions; (3) versatile functionalities, and (4) chiral induction [8]. The combination of boryl reagents, particularly boronate esters, shows advantages of easy accessibility, non-toxicity, air and moisture stability, and a tolerance of many functional groups [9]. The copper-boryl complex is suggested to be the key intermediate for subsequent borylative reactions. The first isolated copper-boryl complex was reported by Sadighi et al. in 2005, in which the Cu-boryl center was stabilized by *N*-heterocyclic carbene (NHC) ligands [10]. This NHC copper boryl complex adopted a linear coordination geometry with the Cu-B distance being 2.002(3) Å; however, for many borylation reactions, the Cu-boryl complexes can be generated in situ by using diboron reagents with a catalytic amount of copper(I) salt. In situ, Cu-boryl-promoted borylation can be traced back to 2000 by Miyaura and Hosomi, who employed a CuCl/KOAc or a copper(I) phosphine catalyst with diboron reagents for conjugate additions to enones, respectively [11,12]. More importantly, boryl reagents have a unique bifunctional characteristic constructed by the Lewis acidic boron atom and the Lewis basic oxygen/nitrogen atoms, which shows an advantage in generating chiral boryl intermediates [13]. Commonly used boron reagents are shown in Figure 1.

**Figure 2:**
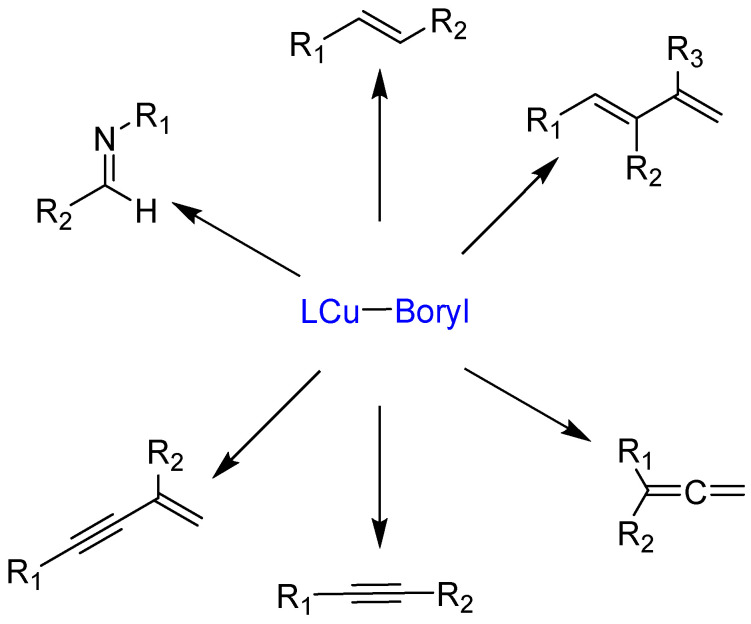
Commonly used substrates for Cu-boryl promoted reactions.

**Scheme 1:**
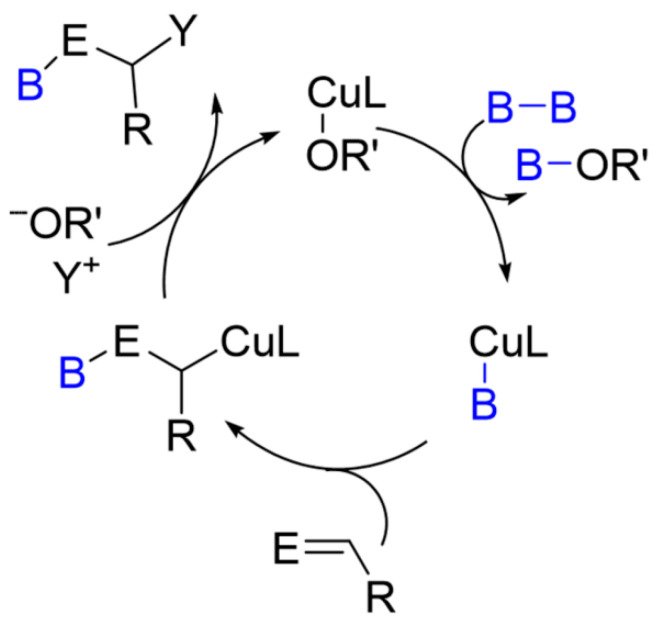
The general mechanism for copper-boryl-mediated reaction with an unsaturated molecule.

The organic synthesis promoted by copper-boryl intermediates has attracted enthusiasm for developing various reactions including hydroboration, carboboration, diboration, and heteroboration with unsaturated substrates of C=C/C≡C/C=E multiple bonds (Figure 2) [14,15,16]. For most of the borylation reactions, the copper alkoxide species is formed in situ by the reaction of copper(I) salt and an alkoxide base [17]. After that, it will react with a diboron reagent to produce the copper-boryl complexes. Finally, the electrophile participates in the reaction. Thus, the ligand exchanges with the alkoxide base and regenerates the copper alkoxide species. The general mechanism is given in Scheme 1 [18].

During recent years, copper-boryl-promoted organic synthesis has advanced comprehensively and rapidly, including in multicomponent reactions, asymmetric synthesis, and the functionalization of reagents [5,19]. Herein, this review will describe the very recent updated progress of the copper-catalyzed borylative functionalization of unsaturated molecules.

## 2. Reaction of Copper-Boryl Species with C-C Multiple Bonds

Alkenes, allenes, and alkynes are widely used in this type of reaction as reactants with copper-boryl intermediates, but control of the regioselectivity in the hydroboration of terminal alkene remains a notorious challenge. Hence, the development of regioselective and stereoselective hydroboration reactions for the non-polar multiple bonds, such as alkenes and alkynes, is highly desired and has attracted numerous interests. This section is mainly focusing on issues with chemo-, regio-, and stereo-selectivity. This has been realized by employing different sets of phosphine and NHC ligands [5].

### 2.1. Hydroboration of Alkenes

**Scheme 2:**
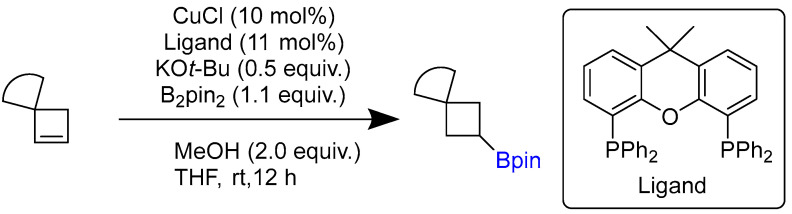
Regioselective hydroborylation of spirocyclobutenes.

The hydroboration reaction allows the formation of valuable organoboron compounds [4]. For the hydroboration of alkenes, Tortosa et al. recently reported a CuCl-catalyzed regioselective borylation of spirocyclobutenes under room-temperature (Scheme 2) [20]. The regioselective transformation mainly depends on the xantphos ligand. By using xantphos and a CuCl catalyst they obtained a single regioisomer with a good yield. Other ligands such as dppp, dppf, BINAP, and dppbz provided a 50:50 mixture of regioisomer. Under the optimized conditions, they synthesized a variety of borylated building blocks with different functional groups such as sulfone, sulfonamide, ether, thioether, difluoromethane, and acetal that are different connectors that can be embedded in the borylated spirocyclic framework. The copper-catalyzed hydroborylation was effectively scaled up to a 6 g scale and obtained an 87% yield [20].

**Scheme 3:**
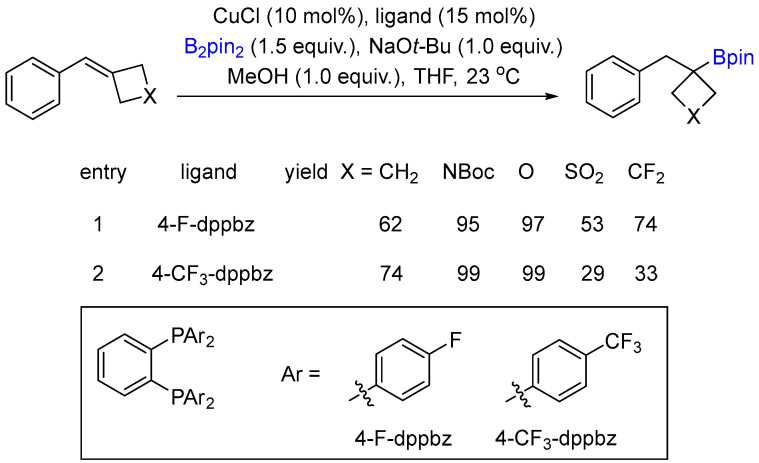
Copper-catalyzed hydroboration of benzylidene-cyclobutanes and related compounds.

McAlpine, Liu, and Engle et al. [21] came up with a method of copper-catalyzed hydroboration of benzylidene four-membered rings to offer synthetically-useful tertiary boronic ester products by using various ligands and reagents with excellent yields of up to 99% (Scheme 3). They also suggested that the roles of ligands in reactions, for example, 4-F and 4-CF_3_-dppbz, were to provide T-shaped π/π interactions among themselves and the substrate. The reactivity was affected mainly by bond interactions between the catalysts and the substrates with varying electronic properties [21].

**Scheme 4:**
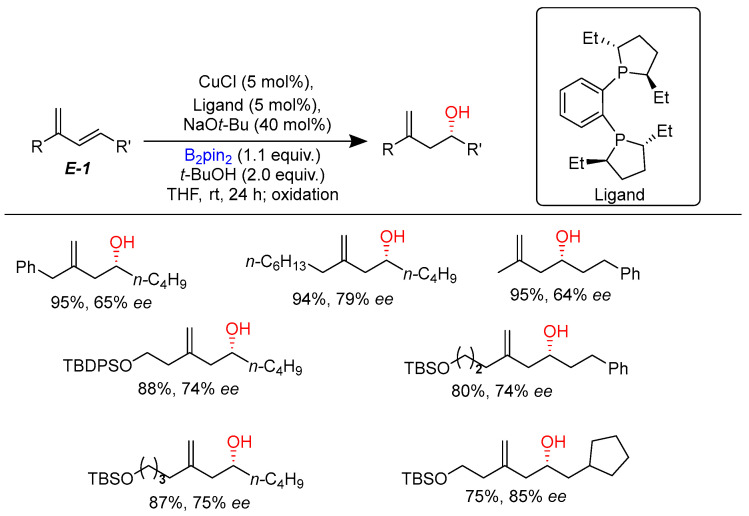
Cu-catalyzed regio- and enantio-selective hydroboration of a disubstituted 1,3-diene.

Diver et al. examined a regio- and enantio-selective Cu-catalyzed hydroboration of 1,3-disubstituted-1,3-dienes (Scheme 4) [22]. The authors utilized the catalytic ene-yne metathesis to attain functionalized 1,3-dienes. The enantio-selective reaction was accomplished by using a chiral ligand (EtDuPhos), whereas a diphosphine 1,2-bis(dipheneylphosphino)benzene ligand was incorporated in achiral reactions. Based on the integrated mechanistic studies, the authors assumed that the reaction went through an allylic copper intermediate.

**Scheme 5:**
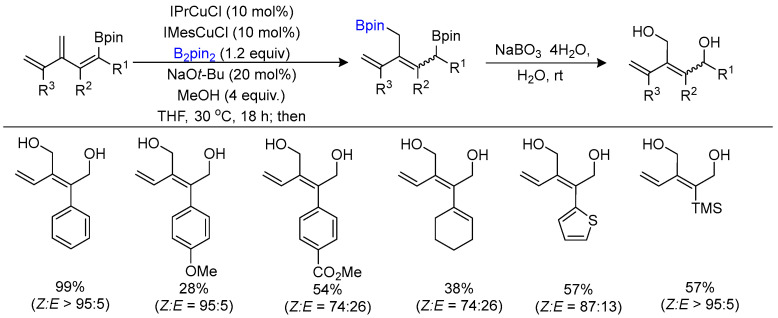
Hydroboration of borylated dendralenes.

In 2020, Fananas-Mastral et al. [23] reported an efficient pathway for the hydroboration of borylated dendralenes which can synthesize functionalized 1,4-addition products by using a NHC-Cu catalyst with good chemo-, regio-, and stereo-selectivity and that can yield up to more than 95% under optimized conditions (Scheme 5). The *Z*-configured diene was attained through a rapid S_E_2 protonation with CH_3_OH, due to the strong stabilization of the allyl/benzyl copper intermediate that causes *E*-selectivity.

**Scheme 6:**
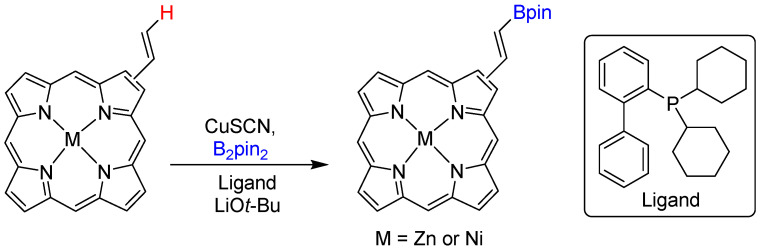
Copper-catalyzed borylation of β-vinyl porphyrinoids.

Specifically, using a non-precious transition metal, Tyurin and Zamilatskov et al. examined the direct C-H borylation of porphyrinoids through a Cu-catalyzed vinylic C-H activation strategy (Scheme 6) [24]. By employing this method, the β- and *meso*-(2-(pinacolboryl)vinyl) porphyrinoids were obtained in good yields with high *E*-stereo-selectivity. Furthermore, the authors studied the synthetic utility of borylated methyl pyropheophorbide-a that was reacted with iodobenzene in the presence of 10 mol% of Pd(PPh_3_)_4_ and three equivalents of Cs_2_CO_3_, resulting in the formation of phenylated derivatives with 50–86% of yields.

**Scheme 7:**
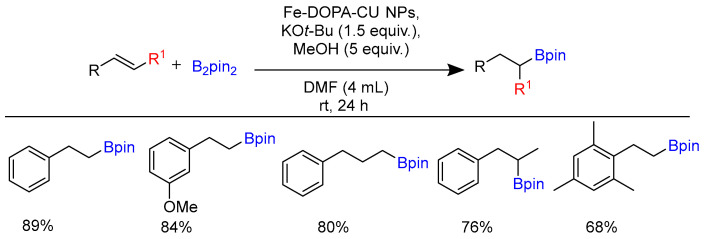
Fe-DOPA-Cu NPs-catalyzed hydroboration of alkenes.

**Scheme 8:**
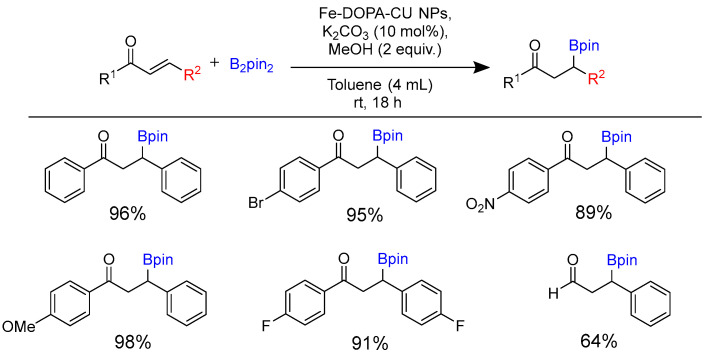
Fe-DOPA-Cu NPs-catalyzed β-borylation of α, β-unsaturated carbonyl compounds.

Besides the development of homogeneous catalytic systems, heterogeneous systems were also developed for reusable catalysts for the hydroboration of alkene. Nano-ferrite-supported Cu-nanoparticles (Fe-dopamine-Cu NPs) as heterogeneous catalysts for the hydroboration of alkenes with B_2_pin_2_ was reported by Kumar Bose et al. (Scheme 7) [25]. Additionally, the same catalyst was utilized for the C-H borylation of benzylideneacetophenone derivatives with B_2_pin_2_ to obtain alkylboronate esters. This protocol tolerated the variety of the functional group (Scheme 8). The catalyst was re-used up to five cycles without a loss of its catalytic activity.

**Scheme 9:**
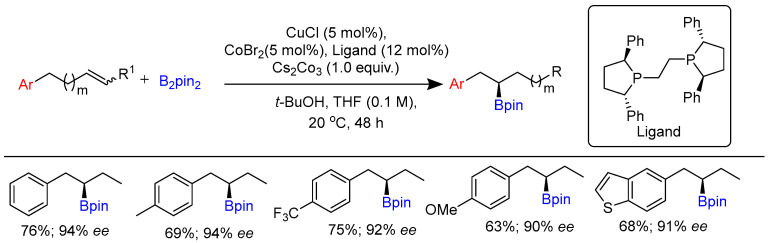
Access to chiral β-alkylboronate through Co/Cu asymmetric relay catalysis.

Xiong et al. developed the bimetallic (Co/Cu) catalytic system for the asymmetric borylation of unactivated alkenes (Scheme 9) [26]. The authors optimized the reaction conditions and found that the use of CoBr_2_ and CuCl as the catalysts with a chiral ligand provided 80% of the target product with 94% *ee*. Using these optimized reaction conditions, and varieties of electron donating with electron withdrawing, the substituents on the aryl ring in alkenes were efficiently transferred in to chiral organoboronates in good yields with enantiocontrol; however, this developed protocol is not suitable for alkene-containing ketones, esters, and amides. The authors performed several mechanistic studies and, based on these results, they suggest that this reaction proceeded through the β-H elimination/olefin insertion endorsed by CoH species produced in situ, followed by Cu-catalyzed asymmetric hydroboration.

**Scheme 10:**
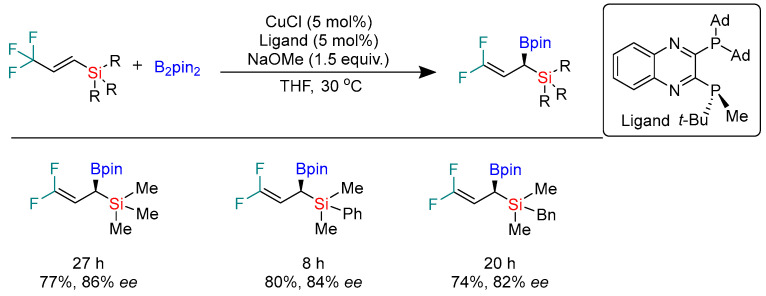
Cu(I)-catalyzed enantioselective γ-borylation of silyl-substituted alkenes.

Using a novel β-fluoroelimination process, the synthesis of optically-active *gem*-difluoro-1-silylallylboronates through the copper-catalyzed enantioselective borylation of trifluoromethyl- and silyl-substituted alkenes using a QuinoxP*-type bisphosphine ligand was disclosed by Ito et al. [27]. The subsequent allylboron compounds went through allylboration with a diverse range of aldehydes to give chiral silyl- and difluoromethylene-containing homoallylic alcohols with a good enantiomeric purity. The mechanism proceeded with the formation of a borylcopper(I) intermediate and the desired product was obtained via a rapid β-fluoroelimination step (Scheme 10).

**Scheme 11:**
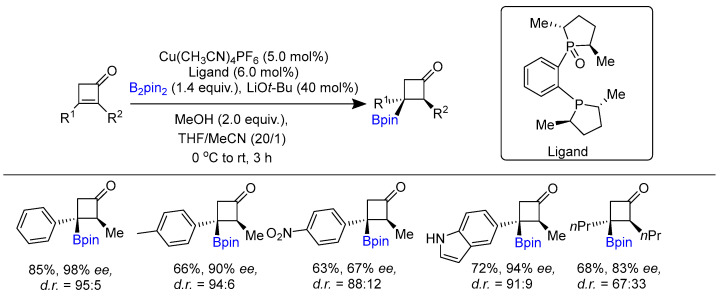
Enantioselective conjugate borylation of cyclobutenones under copper catalysis.

Oestreich et al. [28] identified a chiral bis(phosphine) monoxide (BPMOs) ligand that induced greater levels of enantioselectivity in the copper-catalyzed conjugate borylation of α, β-disubstituted cyclobutenones (Scheme 11). Under the optimized protocol, the substrate scope was investigated by the authors. The electron-rich substituents such as *t*-butyl and methoxy groups reduced the diastereo-selectivity of the target molecule. On the other hand, the electron-withdrawing groups, including CF_3_ and esters, produced the expected products in high enantioselectivities. Advantageously, the 1,6-borylation of *para*-quinone methide also employed this reaction, where it furnished the expected product in a good yield and a moderate enantioselectivity was obtained [28].

**Scheme 12:**
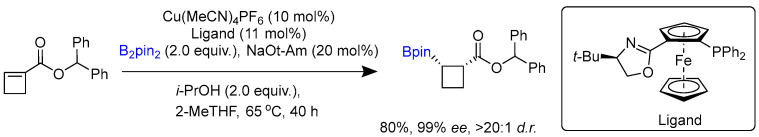
Cu-catalyzed synthesis of a *cis*-β-boronyl cyclobutylcarboxyester.

To obtain chiral cyclobutylboronates, which are valuable synthetic intermediates for the synthesis of bioactive cyclobutanes, Lee and Hall et al. [29] introduced an asymmetric conjugate borylation method via a high throughput ligand screening approach (i.e., 118 ligands). They found that the ferrocene-based ligand could enantio-selectively lead to a *cis*-β-boronyl cyclobutylcarboxyester scaffold with 99% *ee*, and >20:1 *d.r.* The diastereo-selectivity of the copper-catalyzed conjugate borylation could be well rationalized by the quadrant model of the ferrocene-based ligand (Scheme 12).

### 2.2. Hydroboration of Alkynes

**Scheme 13:**
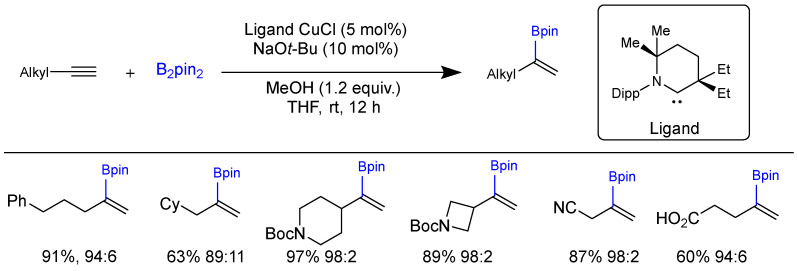
(CAAC)Cu-catalyzed α-selective hydroboration of terminal alkynes.

Bertrand, Jazzer, and Engle et al. [30] found a Cu-catalyzed hydrofunctionalization of alkynes that was enabled by a cyclic (alkyl)(amino)carbene (CAAC) ligand (Scheme 13). Using CAAC/CuCl as the precatalyst, the authors evaluated the scope concerning alkyl-substituted alkynes. By using this developed protocol, electron-rich and electron-deficient substituents, such as halides, COO^−^, CN^−^, and OH^−^ groups, participated in this reaction and provided the expected products in good yields with a high regioselectivity. Upon scaling up the reaction using 5-phenyl-1-pentyne (4.0 mmol) as the substrate, two representative hydroboration reactions proceeded smoothly, furnishing the target product with 81% and 60% yields over two steps [30].

**Scheme 14:**
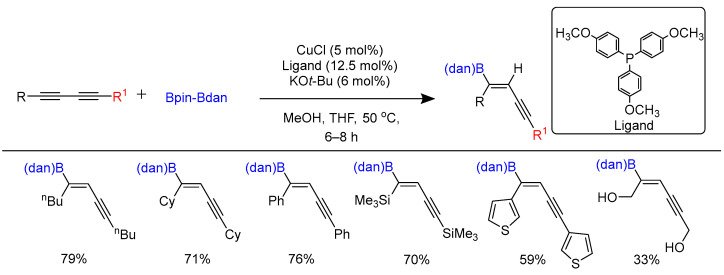
Cu-catalyzed monohydroboration of 1,3-diynes.

Ganesh et al. [31] presented a facile, copper catalyzed, regioselective hydroboration of 1,3-diynes with a Bpin-Bdan reagent to give enynylboronates in a reasonable yield (Scheme 14). The authors identified the suitable reaction conditions to attain 1,4-diboryl,-1,3-dienes in decent yields and regioselectivities. Moreover, the authors executed the synthetic utility of the obtained products by incorporating them in to a Suzuki–Miyaura cross-coupling reaction [31].

## 3. Carboborylation of C-C Multiple Bonds

Carboborylation is another kind of borylation, with some similar characteristics to hydroboration, such as regioselectivity and stereoselectivity, as well as a sensitivity to temperature and solvents [32]. The scope of the carboborylation reaction is large and diverse, including using substrates such as ketones, allenes, alkenes, imines, enones, dienes, etc., whilst using versatile carbo-electrophiles as partners in the reaction. The carboborylation of C=C/C≡C multiple bonds are widely investigated recently.

### 3.1. Carboborylation of Alkenes

**Scheme 15:**
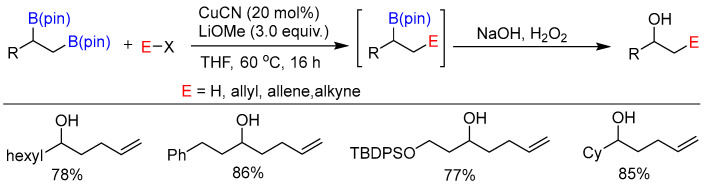
Cu-catalyzed regioselective cross-coupling of alkene.

Following previous work on the Cu-catalyzed borylation of alkenes, Morken et al. [33] developed a Cu-catalyzed coupling of vicinal bis(boronates) with a series of allyl or alkenyl bromides (Scheme 15). Using this operationally simple method, the authors obtained a wide range of terminal alkenes in good yields. This developed catalytic strategy delivered the target molecules in high regioselectivity. On the basis of DFT studies, the authors identified that the key role was played by the cyclic ate complexes [33].

**Scheme 16:**
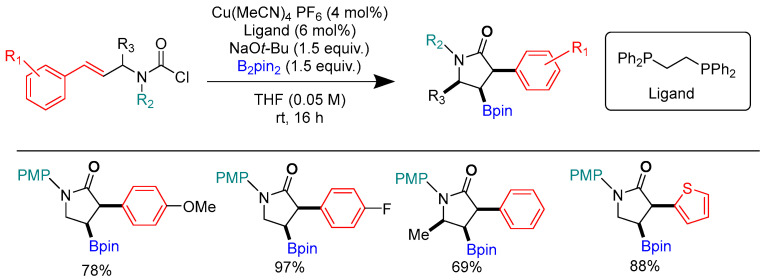
Copper-catalyzed diastereoselective borylacylation of 1,2-disubstituted alkenes.

For the synthesis of chiral borylated γ-lactams, Lautens et al. [34] have shown the copper-catalyzed intramolecular borylacylation of either 1,2- or 1,1-disubstituted alkenes with a tethered carbamoyl chloride (Scheme 16). The authors explored the substrates’ scope of styrenyl substituents by utilizing various electron-donating and electron-withdrawing groups, with 3,4-disubstituted γ-lactams having electron-donating groups on the *para* position giving the lower yield; however, 3,4-disubstituted γ-lactams having electron-donating groups provided a higher yield. The asymmetric borylacylation of 1,1-disubstituted alkenes was performed under optimal conditions, and the mechanism of the reaction proceeded through the *S*-configured benzyl-copper intermediate. A chiral borylated γ-lactam ring was formed via the cyclization of a benzyl-copper intermediate. [34].

**Scheme 17:**
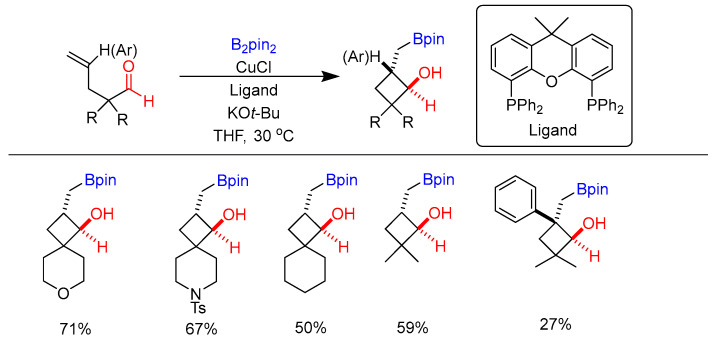
Copper-catalyzed borylative ring closing reaction.

The copper-catalyzed borylative ring closing of unactivated alkenes bearing electrophilic sites involves a strategic intramolecular 1,2-carboborylation process and was reported by Carbo and Fernandez et al. [35]. They achieved the copper(I) catalyzed borylative cyclization method to synthesize *anti*-diastereoselective 2-(borylmethyl)cycloalkanols with γ-alkenyl aldehydes as the starting materials. This reaction occurred through a regioselective boryl addition to the C=C bond followed by an intramolecular 1,2-addition of the Cu-C bond onto the C=O bond (Scheme 17).

**Scheme 18:**
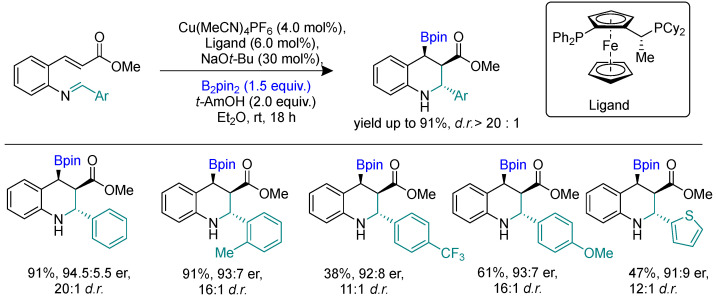
Enantio- and diastereo-selective conjugate borylation/Mannich cyclization.

In 2021, Lautens et al. [36] documented a protocol to synthesize enantioenriched *N*-heterocycles through a copper-catalyzed conjugate borylation or a Mannich cyclization (Scheme 18). This reaction was first published to generate imines with high enantioselectivity under mild conditions. Strategies to capitalize on the enolate intermediates to α, β-unsaturated carbonyl systems are rare, while the few examples existing are limited to either being non-enantioselective or intermolecular. This catalytic system is easy to handle, is readily open to scale-up and creates complex *N*-heterocycles with stereocenters. Furthermore, several Michael acceptors illustrate that this method is widely available (Scheme 18).

**Scheme 19:**
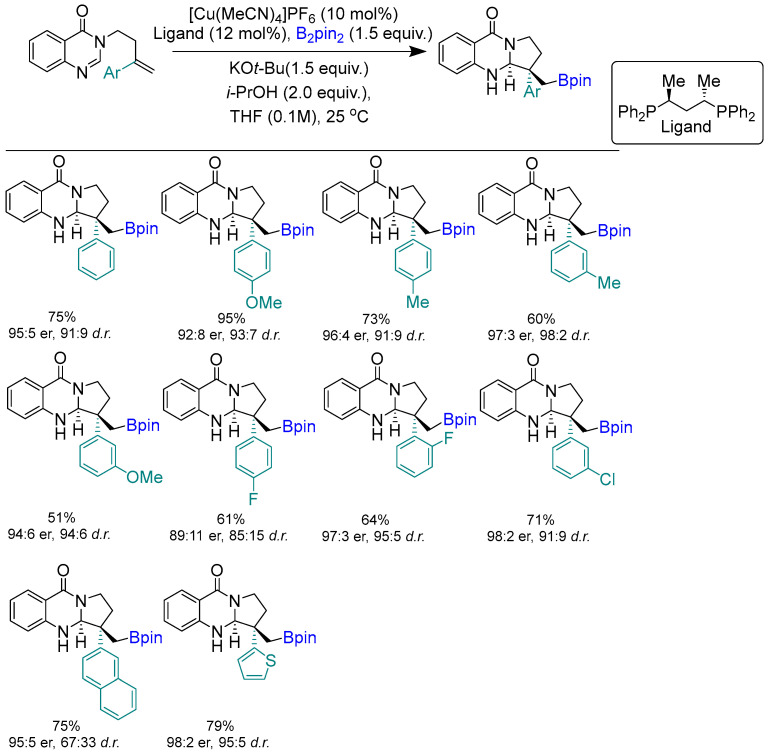
Scope of Cu-catalyzed borylative cyclization for the synthesis of pyrroloquinazolinone.

In the same year, Procter et al. [37] published the enantioselective synthesis of pyrroloquinazolinone via copper-catalyzed borylative cyclization (Scheme 19). A higher yield of the desired product was obtained when using Cu(MeCN)_4_PF_6_ with KO*t*-Bu in THF. This strategy allowed various aryl-substituted alkenes to proceed efficiently and deliver pyrroloquinazolinones with good to excellent enantio- and diastereo-control. The authors proposed a mechanism for this reaction that proceeded through the formation of copper-boryl species as one of the key intermediates [37].

**Scheme 20:**
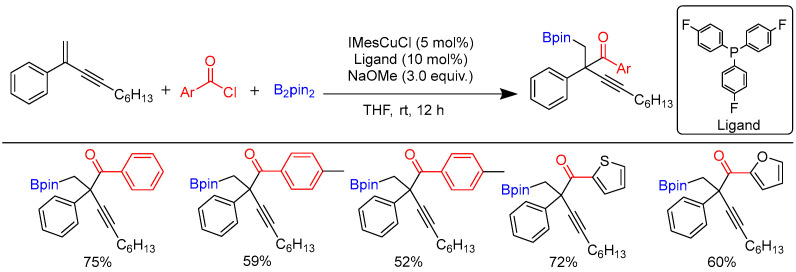
Copper-catalyzed 1,2-borylacylation of 1,3-enynes.

The borylacylation of alkenes is an important and efficient method for the rapid synthesis of carbonyl compounds with structural diversity. For example, Zhang and Peng et al. [38] recently reported a copper catalyzed 1,2-borylacylation of 1,3-enynes with B_2_pin_2_ and acid chlorides in 2022 (Scheme 20). After screening several ligands, they obtained good results with a P(4-FPh)_3_ ligand. The reaction proceeded with a range of highly functionalized α,α-disubstituted β-alkynyl ketones that were readily synthesized under mild conditions in moderate to good yields with high regioselectivity. Furthermore, the authors treated the products with NaBO_3_, while 4H_2_O provided 1,2-allenyl ketones, which was anticipated to proceed via a retro-aldol process of the corresponding homopropargyl alcohols. The authors suggested that the reaction proceeded via a borylated allenyl-copper intermediate [38].

**Scheme 21:**
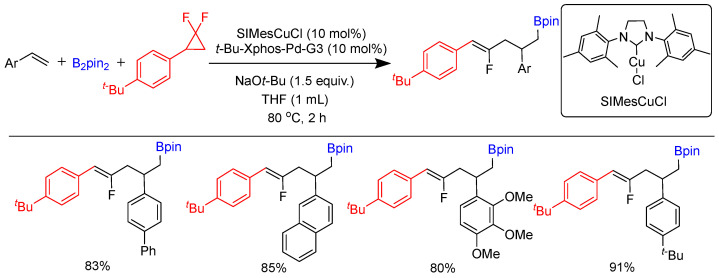
Three-component reaction of alkenes for the synthesis of boryl-substituted fluoroalkenes.

The exploration of multicomponent reactions to install boron and fluorine-containing fragments into alkenes is highly desired. In this regard, Gong and Fu, et al. [39] investigated the first example of dual Cu/Pd catalyzed borylfluoroallylation of alkenes via the C(sp^3^)–C(sp^3^) coupling of *gem*-difluorinated cyclopropane with alkenes and B_2_pin_2_ (Scheme 21**)**. The authors were systematically optimizing the reaction conditions and they obtained a high yield of the expected product by using a SIMesCuCl and *t*-Bu-Xphos-Pd-G3 catalytic system. The scope of alkenes and *gem*-difluorinated cyclopropanes was explored. In all the attempts, the expected alcohols were obtained in good yields and heterocyclic compounds were easily incorporated into this reaction and afforded the expected product in a good yield. A gram-scale synthesis (up to 3 mmol) of this protocol was conducted and they obtained 92% of the yield. In order to elucidate the mechanism of the three-component coupling reaction, a model reaction was performed by using an alkyl diborate reagent under the optimized conditions. In this case, the authors did not obtain the target product. Based on this result, the authors confirmed that the diboron compound was not an intermediate for the three-component reaction [39].

**Scheme 22:**
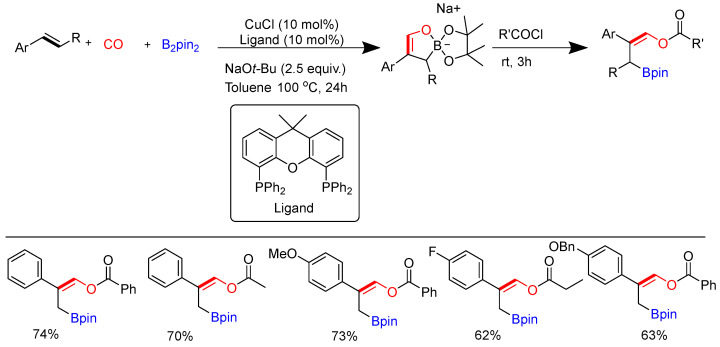
Synthesis of β-boryl vinyl esters.

Wu et al. [40] developed a strategy for the synthesis of sodium cyclic borate intermediates through the one-pot borofunctionalization of styrenes, B_2_pin_2_, CO, and NaO*t*-Bu in the presence of xantphos/CuCl as a catalyst (Scheme 22). By using this developed protocol, they utilized CO as the C1 source to easily prepare a multifunctional β-boryl vinyl ether, β-boryl aldehydes, β-boryl carbonates, and β-boryl vinyl esters. In addition, nerol, (−)-borneol, (−)-menthol, diacetonefructose, 1,2:3,4-di-O-isopropylidene-α-d-galactopyranose, and 5α-cholestan-3β-ol derived styrenes participated this reaction successfully, furnishing the products in good yields.

**Scheme 23:**
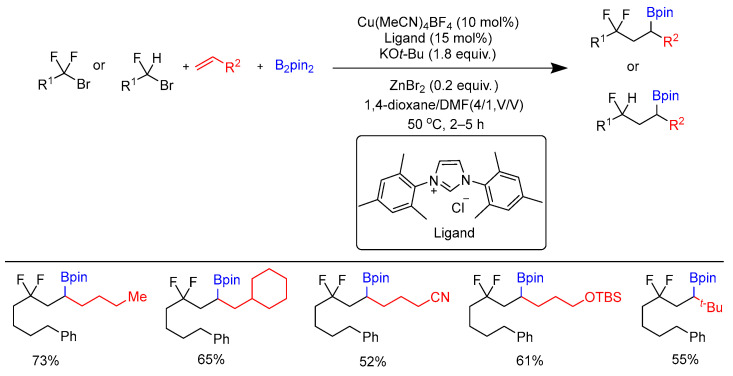
Cu-catalyzed intermolecular 1,2-alkylborylation of olefins.

Ito et al. [41] launched the intermolecular 1,2-alkylborylation of unactivated olefins that proceeded via a radical-relay mechanism (Scheme 23). Initially, the authors screened the reaction conditions and found that the desired product was obtained by using a [Cu(MeCN)_4_]BF_4_/ligand catalyst system with K(O*t*-Bu), and a catalytic amount of ZnBr_2_ as an additive in 1,4-dioxane/DMF (4/1, *v/v*) at 50 °C. Using the optimized conditions, the authors further investigated the scope of mono- and di-fluoro alkyl bromides. All the substrates underwent this reaction successfully and gave the target product in a good yield. Nevertheless, aromatic olefins did not furnish this reaction under the optimal reaction conditions and this may be due to the copper(I)-boryl intermediate first reacting with the aromatic alkene rather than with the alkyl bromide [41].

**Scheme 24:**
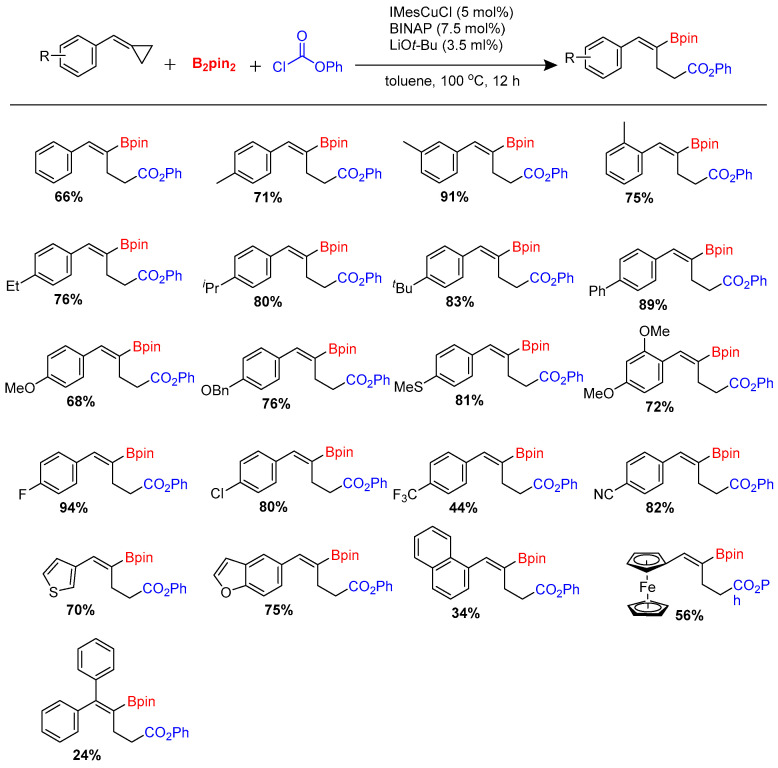
Substrate scope of BCPs.

Peng et al. [42] synthesized γ-boryl-γ, δ-unsaturated carbonyl compounds through the copper-catalyzed borocarbonylation of benzylidenecyclopropanes (BCP) via a proximal C-C bond cleavage. By using this methodology, a broad range of γ-boryl-γ, δ-unsaturated esters were prepared with excellent regio- and stereo-selectivity (Scheme 24). This developed catalytic methodology involved the cleavage of a C=C bond and the formation of new C-C and C-B bonds.

**Scheme 25:**
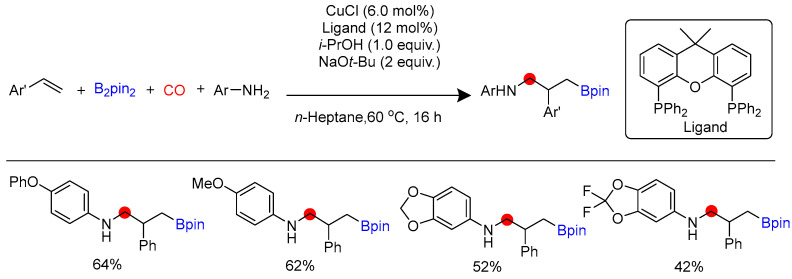
Boroaminomethylation of olefins.

**Scheme 26:**
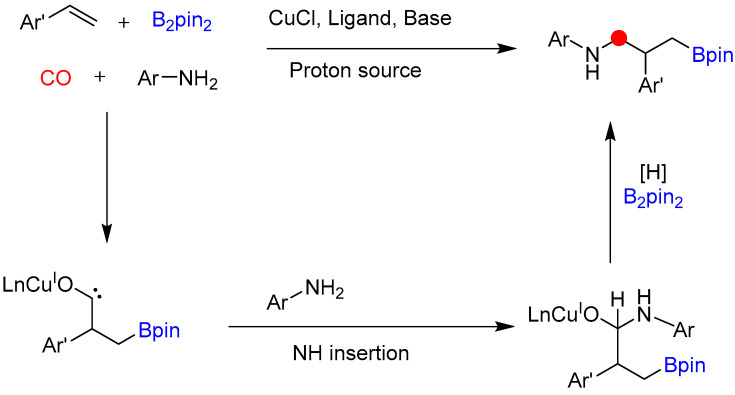
Mechanism of broaminomethylation of olefins.

The carbonylative aminomethylation using carbon monoxide (CO) as a C1 building block was reported by Wu et al. [43] to execute a copper-catalyzed boroaminomethylation of olefins via a four-component coupling reaction to achieve γ-borylamines (Scheme 25). This catalytic process allowed a vast range of functional group tolerance and afforded the γ-boryl amines in reasonable yields. The non-requirement of an additional reducing agent to reduce the CO is the added advantage of this methodology. This reaction pathway occurred via an in situ tailored carbene intermediate insertion into the amine N-H bond followed by a borylation reaction. (Scheme 26). The authors performed ^13^C labeling studies in order to confirm that the “C’’ in the CH_2_ group was raised from one molecule of CO. The obtained products through this reaction were further transformed into high-value compounds including aromatization to afford quinolines [43].

**Scheme 27:**
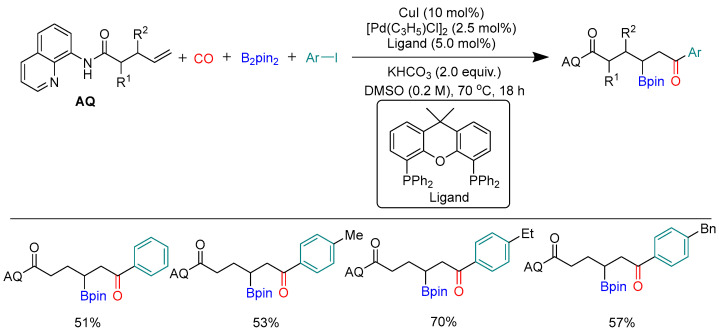
Pd/Cu-catalyzed borocarbonylation of unactivated alkenes.

**Scheme 28:**
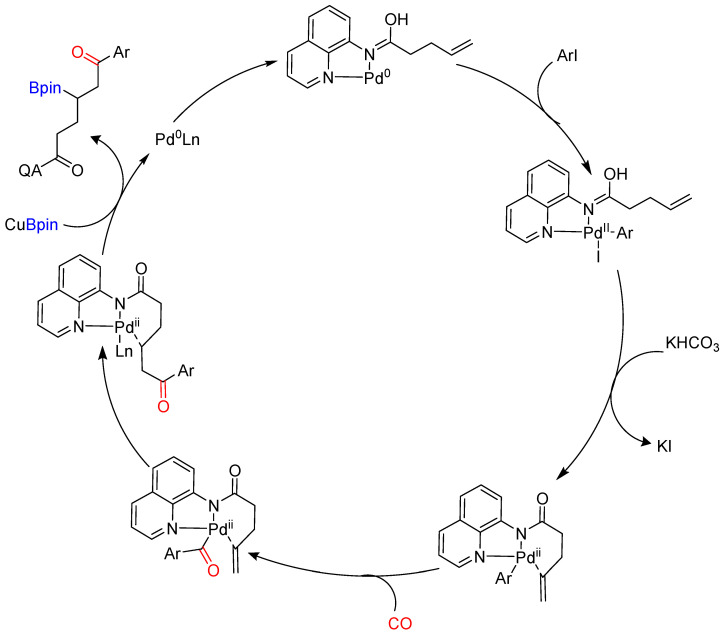
Mechanism for Pd/Cu-catalyzed borocarbonylation of alkenes.

Wu et al. [44] developed the borocarbonylation of alkenes through cooperative Pd/Cu catalysis (Scheme 27). For this reaction, an 8-aminoquinoline directing group and slower CuBpin formation by using KHCO_3_ were important for a successful reaction design. Different substituted aryl iodides and aliphatic alkenes were transformed into the desired β-boryl ketones in moderate to excellent yields; however, no desired products were obtained in the case of 3-butenoic amide, 2-vinylbenzamide, or internal alkene tested under the optimized conditions [44]. The proposed mechanism involves the 8-aminoquinoline directing group coordinating with Pd(0) (Scheme 28), which promotes the oxidative addition of aryl iodides to generate aryl iodo Pd(II). After a ligand exchange, the CO insertion leads to the acyl-Pd(II) species, which undergoes a migratory insertion with alkene to generate a C(sp^3^)-Pd(II) intermediate. Subsequently, the C(sp^3^)-Pd(II) complex reacts with CuBpin to give the product, β-boryl ketone, and to regenerate the Pd(0) complex (Scheme 28) [44].

**Scheme 29:**
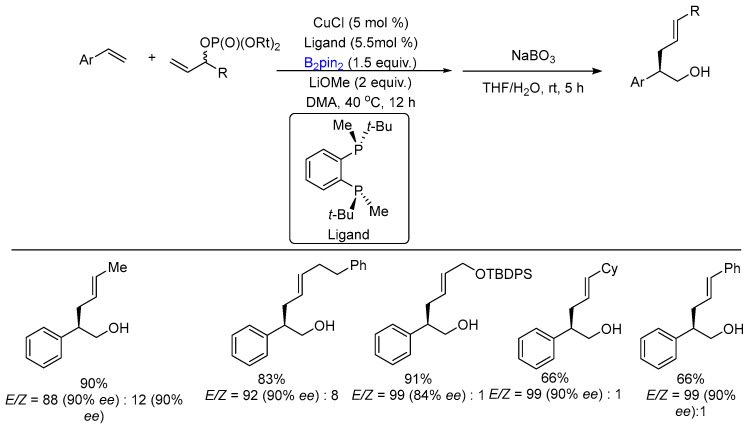
Direct allylation of chiral alkylcopper nuclophiles.

In 2020, Baik and Yun et al. [45] found a Cu-catalyzed stereoconvergent coupling reaction of vinyl arenes and racemic acyclic allylphosphates with B_2_pin_2_ (Scheme 29). Using this methodology, a new stereoselective C-C bond was formed. Furthermore, the authors explored the scope of vinyl (hetero) arenes and 2° allylic phosphates bearing alkyl and phenyl substituents and these reactions provided the expected products with higher enantioselectivities up to 95% *ee*.

**Scheme 30:**
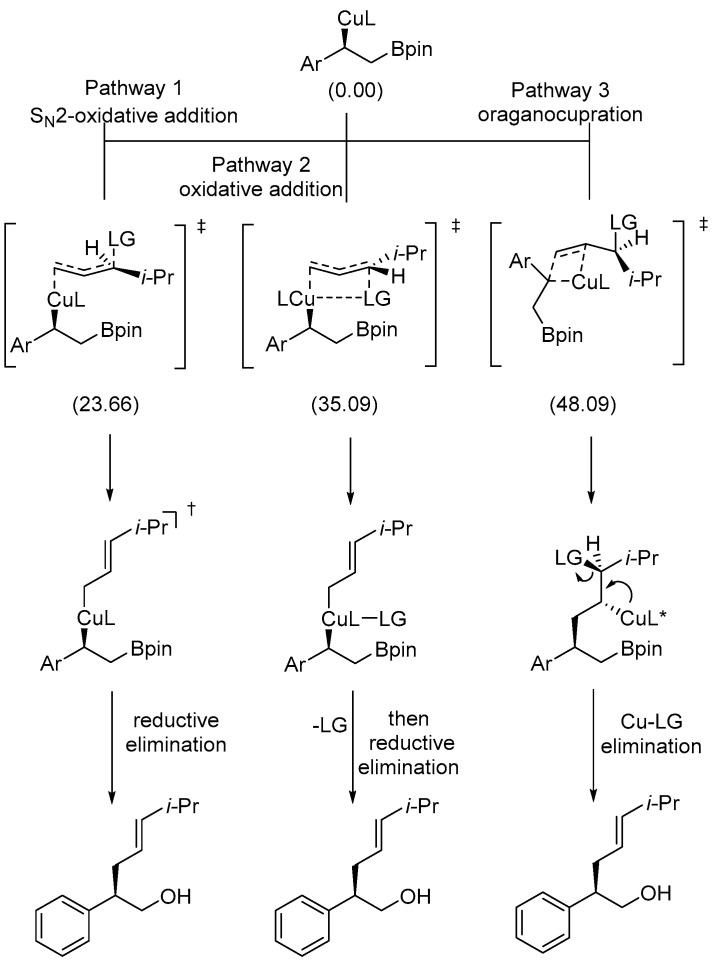
Three possible reaction pathways with DFT calculation results (energy data in kcal/mol).

According to the DFT calculation result, three possible reaction pathways were considered for the allylic substitution of the organocopper intermediate, i.e., the S_N_2-oxidative addition (pathway 1) toward the allyl phosphate with the simultaneous dissociation of a leaving group, the oxidative addition pathway with the association of the cupper center and a leaving group (pathway 2), or a stepwise organocupration followed by the β-elimination of a copper complex (pathway 3) (Scheme 30) [45].

**Scheme 31:**
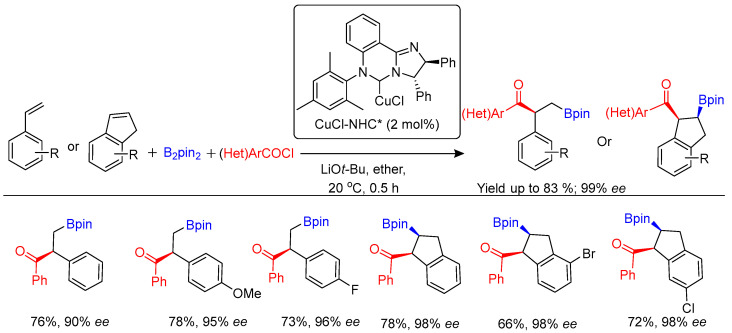
Cu-catalyzed asymmetric borylacylation of styrene and indene.

Lu and Li et al. [46] showcased the enantioselective copper-catalyzed borylacylation of aryl olefins with acyl chlorides and bis(pinacolato)diboron (Scheme 31). The merits of this reaction are (i) a low catalyst loading (2 mol%), (ii) a shorter reaction time and (iii) at room-temperature. The reaction can be scaled up and the β-borylated ketone products could undergo several further transformations.

### 3.2. Carboborylation of Allenes

The borylative functionalization of allenes catalyzed by copper with diverse electrophiles, including alkyl halides, aldehydes, and ketones, has become a predominant tool to develop complex molecular architectures [6]. From allenes, allylic boronates can be constructed as important building blocks in synthetic organic chemistry on account of their synthetic utility, high thermal stability in terms of the C-B bond, and nontoxicity [5,6].

**Scheme 32:**
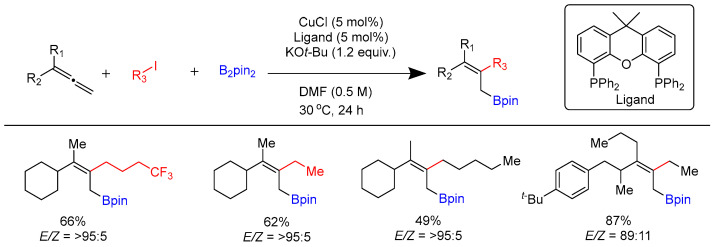
Allylic boronate-selective carboborylation reaction of *gem*-alkylallenes.

A challenging intermolecular three-component coupling reaction of allenes, alkyl halide, and a diboron reagent in the presence of a copper catalyst was reported by Ito et al. [47] to construct straightforward access to multi-alkylated allylic boronates (Scheme 32). The allylboration of aldehydes allows the diastereoselective construction of quaternary carbon atoms. The stereoselectivity mechanism of this multicomponent reaction was elucidated by using DFT calculations. This reaction was extended to the different substrates of *gem*-dialkylallenes [47].

**Scheme 33:**
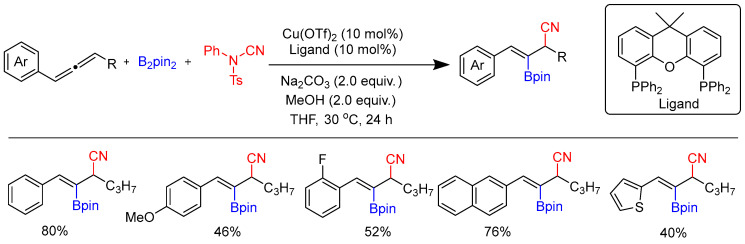
Copper-catalyzed regiodivergent cyanoboration of allenes.

Jiang and Liu et al. disclosed a Cu-catalyzed cyanoboration of allenes with B_2_pin_2_ and NCTs (*N*-cyano-*N*-phenyl-*p*-toluenesulfonamide). From this reaction, the authors obtained cyanoborylated products with higher regio- and stereo-selectivity (Scheme 33) [48]. They found that the use of Cu(OTf)_2_ (10 mol%), a ligand (10 mol%), Na_2_CO_3_ (1.2 equiv), and MeOH (2.0 equiv) in THF at 30 °C provided a single isomer. Furthermore, the bidentate ligands with large bite angles enhanced the regioselectivity and the yield of the product as well. In addition, derivatizations of the cyanoborylated products were established by using different varieties of allenes and provided the target products with acceptable yields. On the basis of DFT calculations and experimental observations, the rate-determining step of transmetalation with B_2_pin_2_ was suggested [48].

**Scheme 34:**
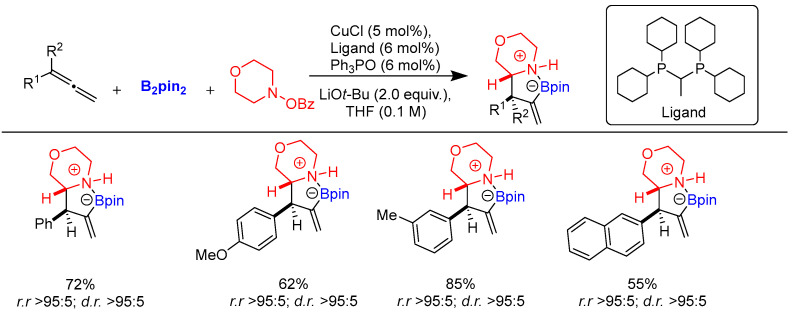
Borylative α-C-H allylation and their key mechanistic aspects.

The three-component carboborylation of allenes typically involves several challenges associated with the regio- and stereo-selective generation of the catalytic Bpin-substituted allyl copper intermediate. In connection with this, Fañanás-Mastral et al. [49] identified a method to obtain α-functionalized cyclic secondary amines from *O*-benzoyl hydroxylamine through a Cu-catalyzed coupling reaction of allene and B_2_pin_2_ (Scheme 34). Due to the addition of a catalytic amount of Lewis base into this reaction, the authors observed higher chemoselectivity.

**Scheme 35:**
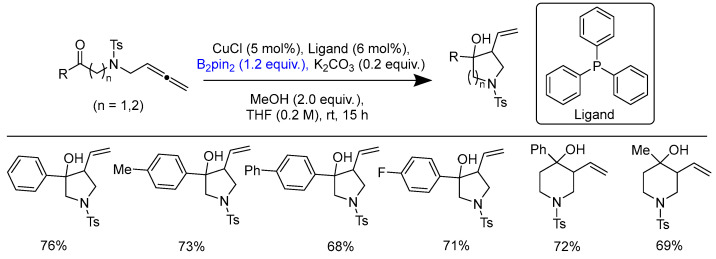
Cu-catalyzed reductive cyclization of ketoallenes.

The diastereoselective Cu-catalyzed reductive intramolecular cyclization of allene-tethered ketoamine was recently reported by Cho et al. (Scheme 35) [50]. This reaction progressed via a borylative allyl copper intermediate formed from allenes and this intermediate underwent intramolecular diastereoselective cyclization followed by cascade copper-catalyzed hydrodeborylation, to give 3-hydroxypyrrolidines in good yields.

**Scheme 36:**
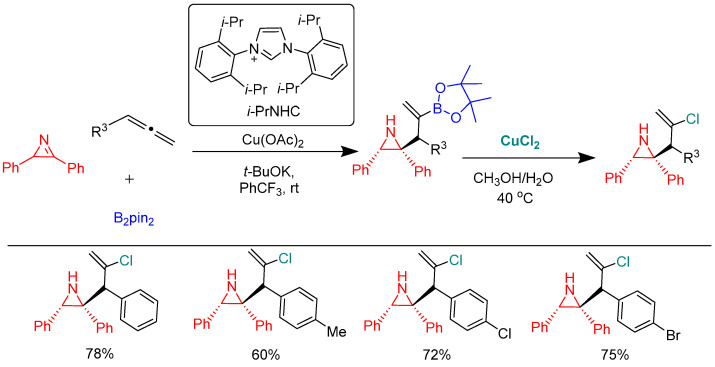
Cu-catalyzed three-component carboboronation of allenes.

Zhang et al. [51] used 2H-azirines as the electrophile for the three-component carboboration of arylallenes catalyzed by an NHC-Cu catalytic system (Scheme 36). The reaction exhibited excellent diastereoselectivity and good yields. The borylated products were further chlorinated by using CuCl_2_. This catalytic approach was further extended to different azirines, alky and aryl allenes. A scaled-up (up to 1 mmol) synthesis was then evaluated by the authors and obtained 61% of the yield.

### 3.3. Carboborylation of Alkynes

**Scheme 37:**
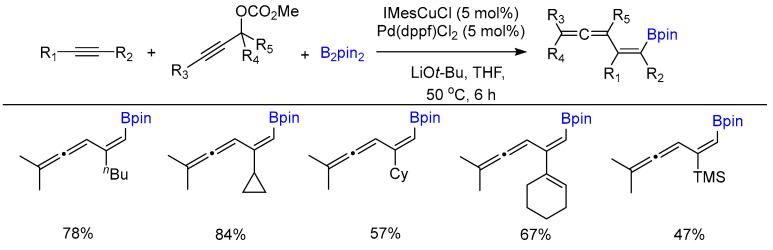
Cu and Pd-catalyzed carboborylation of alkyens.

Unsaturated alkynes with a C≡C triple bond offer similar characteristics to alkenes, dienes, and allenes, thus, the borylative difunctionalization of alkynes has emerged as a powerful approach to the synthesis of highly functionalized alkenes. Gong and Fu et al. [52] put forward a method catalyzed by Cu/Pd to synthesize densely (i.e., tetra-, penta-, and hexa-) substituted ene-allenes in an acceptable yield using readily available reagents such as propargylic carbonates and bis(pinacol)diboron, which had high stereoselectivity and high regioselectivity (Scheme 37) [52].

**Scheme 38:**
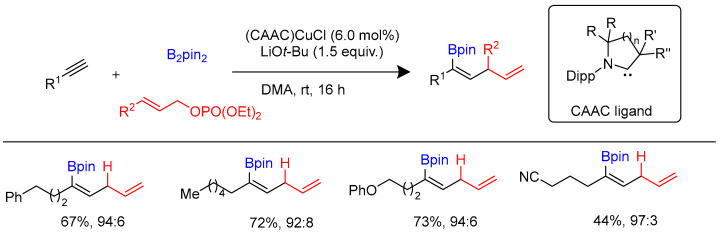
α-selective allylboration of terminal alkynes.

Recently, Bertrand, Jazzar, and Engle et al. [53] demonstrated a regioselective three-component carboborylation of terminal alkynes through cyclic alkyl amino carbene (CAAC) copper catalysis (Scheme 38). A series of CAAC·CuCl catalytic systems with different steric and electronic properties were tested. The authors found that an ethyl substituted EtCAAC5-ligated Cu catalytic system (CAAC ligand with CuCl) promoted transformations with both a high conversion and high α-selectivity. The replacement of the ethyl substituent with either an electron-withdrawing group or more sterically-bulky groups led to a decreased yield and α:β ratio. Various substitutions on the terminal alkynes and alkyl iodides were well tolerated in this reaction and delivered the expected products in good yields with high regioselectivity. The authors proposed a mechanism through a reversible borylcupration that accounted for the change in regioselectivity as a function of the electrophile identity [53].

**Scheme 39:**
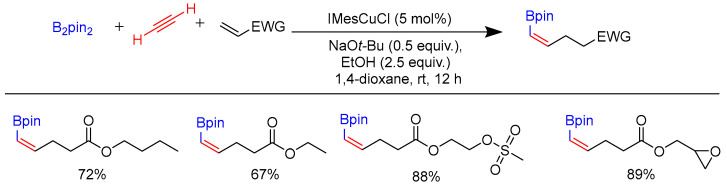
Cu-catalyzed carboborylation of acetylene with B_2_pin_2_ and Michael acceptors to provide alkenyl boronates.

Zhu et al. [54] portrayed a copper-catalyzed carboborylation of acetylene with B_2_pin_2_ and Michael acceptors (Scheme 39). This catalytic approach is successful for the substrates such as acrylates, thioacrylates, acrylonitrile, vinyl ketones, and vinyl sulphones and it afforded the target molecules with decent yields. This transformation was successfully applied in a large-scale synthesis [54].

**Scheme 40:**
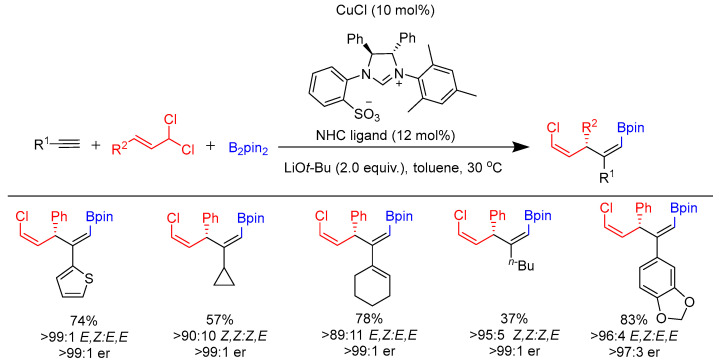
Copper-catalyzed allylboration of alkynes with allylic *gem*-dichlorides.

Fañanás-Mastral et al. [55] reported the copper-catalyzed allylboration of alkynes in 2022, using *gem*-dichlorides to obtain difunctional *E,Z*-dienes in good yields (Scheme 40). The combined Cu/NHC ligand catalytic system was robust and delivered the target molecules with enantio- and diastereo-selectivity. The in situ generated complex of an NHC ligand with a Li cation played a vital role in the stereo-control of this reaction, and this fact was supported by DFT calculations [55].

**Scheme 41:**
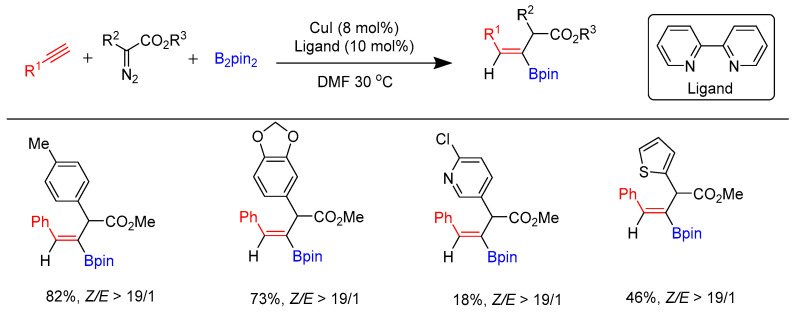
Cu-catalyzed borylative alkylation of terminal alkynes.

Sun et al. [56] recognized a route to access alkenyl boronates via a copper-catalyzed three-component reaction of alkynes, B_2_pin_2_, and a diazo compound (Scheme 41). The authors found that the combination of CuI with a bipyridine ligand yielded 88% of the expected product. The scope of different alkynes and diazo compounds was explored and all the reactants successfully participated in this reaction with moderate to good yields. Furthermore, the synthetic utility of this boroalkylation of terminal alkynes was demonstrated by conducting versatile derivatization reactions of Suzuki–Miyaura cross-coupling. This three-component reaction was supposed to be initiated by the formation of a copper acetylide intermediate from the copper catalyst and phenylacetylene [56].

**Scheme 42:**
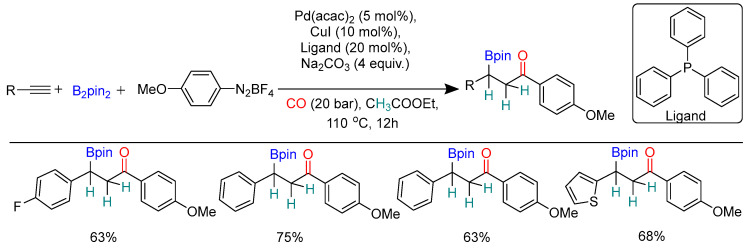
Pd/Cu-catalyzed carbonylation and borylation of alkynes.

The carbonylative borylation of alkynes is one of the important transformations in the transition-metal-catalyzed borofunctionalization of alkynes. For example, Wu et al. [57] established a cooperative Pd/Cu-catalysis for the multicomponent carbonylation and borylation of alkynes (Scheme 42). This methodology was successfully extended to varieties of aryl alkynes and obtained the expected molecules in good yields. Aromatic/aliphatic diynes, internal alhynes, 3-methyl-1-butyne, and 3-phenyl-1-propyne, were unsuccessful for this approach. Preliminary mechanistic studies revealed that the three hydrogen atoms of the product originated from ethyl acetate [57].

**Scheme 43:**
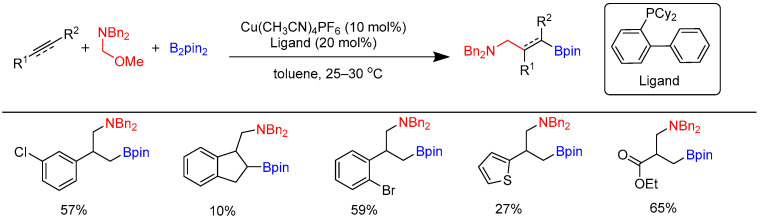
C-C double and triple bonds in the copper-catalyzed borylaminomethylation with *N*,*O*-acetals.

Tao et al. [58] reported a borylative aminomethylation of alkene and alkynes with B_2_pin_2_ (Scheme 43). The combined use of Cu(CH_3_CN)_4_PF_6_ and Cy-JohnPhos as a ligand efficiently converted the alkenes and alkynes in to the amino borylated products in good yields. This catalytic protocol was robust and it was easy to access the γ-aminoboronates. Notably, 67% of the target product was obtained in the case of a bulk scale synthesis. [58].

### 3.4. Carboborylation of Imine and Carbonyl Derivatives

**Scheme 44:**
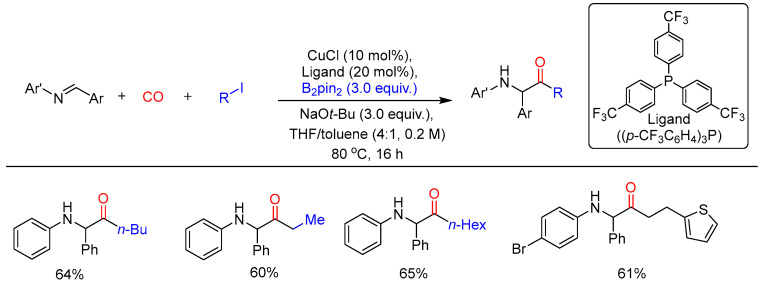
Strategies for regiodivergent borocarbonylation of imines.

Recently, Wu et al. [59] explored the possibility of incorporating imine and alkyl iodides for the synthesis of α-boryla amides and α-amino ketones. The regioselectivity problem was overcome by utilizing a (*p*-CF_3_-C_6_H_4_)P ligand; however, they could obtain high regioselective-corresponding a-boryl amides, while MeIMes was used as the ligand (Scheme 44) [59].

**Scheme 45:**
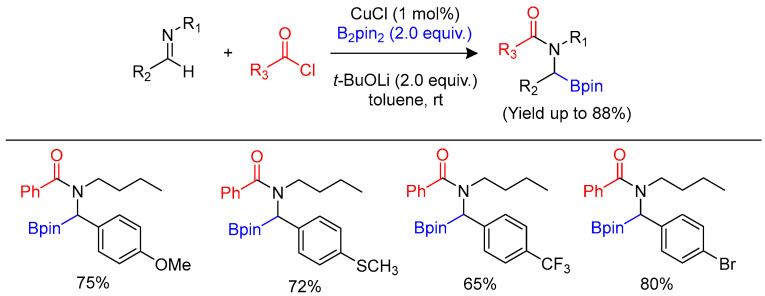
Cu-catalyzed 1,2-addition of imines.

Song et al. [60] reported a developed method of tunable synthesis of α-amino boronic esters using the available aldehydes and amines via copper-catalyzed borylacylation, which had an excellent functional group tolerance with yields up to 88% among 37 examples (Scheme 45).

**Scheme 46:**
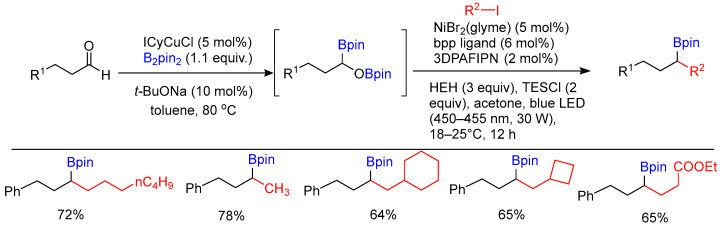
Alkylboration of aldehydes catalyzed by copper and nickel.

A novel route to attain secondary α,α-dialkyl boronates via the catalyzed copper deoxygenative alkylboration of aldehydes was identified by Xu et al. (Scheme 46) [61]. The desired product in a high yield was achieved by using the following conditions of Ni (5 mol%), and a bpp ligand (6 mol%) in toluene. The authors were unsuccessful while using tertiary aliphatic aldehyde and aromatic aldehyde [61]; hence, this approach is limited for aliphatic aldehydes only.

## 4. Heteroboration of C-C Multiple Bonds

**Scheme 47:**
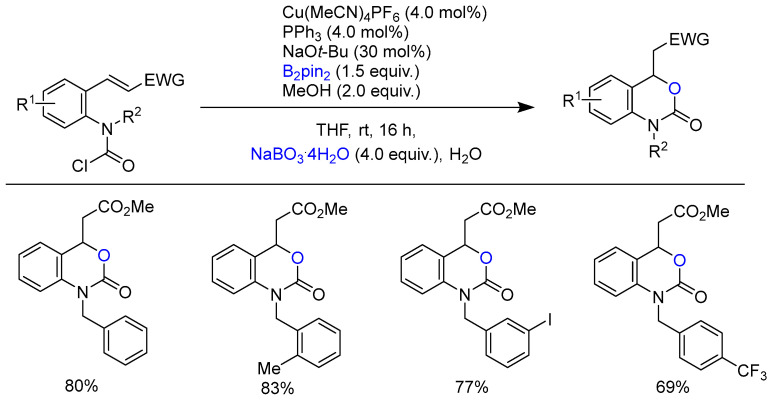
One-pot synthesis of benzoxazinones using B_2_pin_2_.

Some other reactions are limited in application and number, nevertheless, they do have a place in borylation and they represent significant pieces of boration; however, unfortunately, it seems that this kind of reaction has ground to a standstill due to some unknown reasons. For example, Lautens et al. [62] used a Bpin group as a pronucleophile to synthesize benzoxazinone in a one-pot synthesis (Scheme 47). This one-pot operation involved an initial copper-catalyzed borylation and subsequent C-B bond oxidation to generate the reactive alcohol intermediate followed by cyclization. The developed one-pot, three-step method was mild and could be applied to synthesize a broad range of benzoxazinone scaffolds, while it also could be applied to enantioselectivity.

**Scheme 48:**
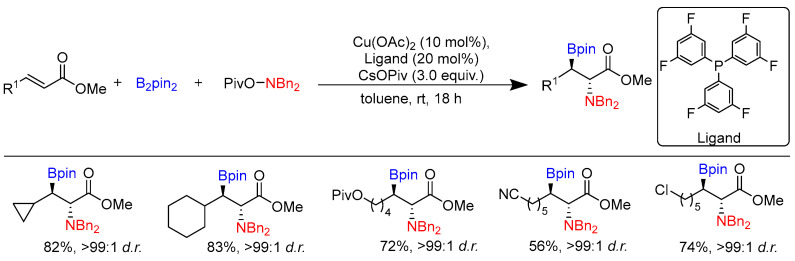
Cu-catalyzed aminoboration of α, β-unsaturated esters.

Very recently, Hirano et al. [63] demonstrated a catalytic way to the synthesis of acyclic *anti-*β-boryl-α-amino acids, with a high *anti*-diastereoselectivity (up to >99:1) via a copper-catalyzed aminoboration of α, β-unsaturated carboxylic acid derivatives with B_2_pin_2_ and hydroxylamines (Scheme 48). This finding offered a facile route to access β-boryl amino acid derivatives [63].

**Scheme 49:**
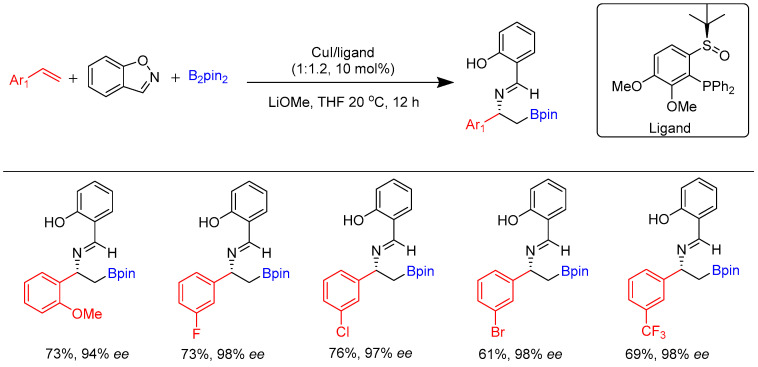
Aminoboration of alkenes using Cu as a catalyst.

In order to synthesize the chiral β-aminoboronate from an easily accessible method, Jian et al. [64] developed the enantioselective aminoboration of styrenes with 1,2-benzisoxazole as a nitrogen source by using a copper-chiral sulfoxide-phosphine ligand as a catalytic system (Scheme 49). Under this approach, a variety of β-aminoboronates were prepared in good yields.

## 5. Multiboration

**Scheme 50:**
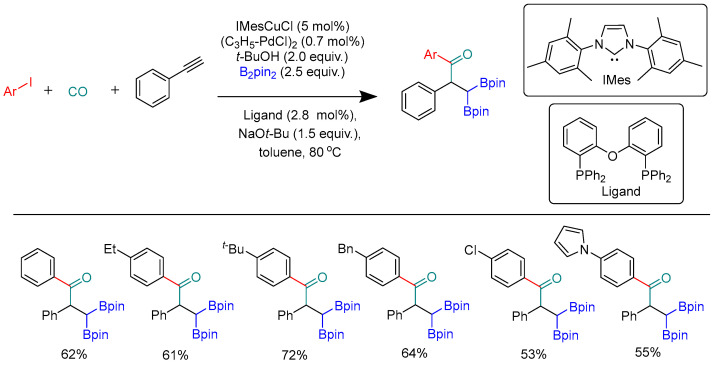
Cu/Pd-catalyzed borocarbonylative trifunctionalization of alkynes.

Multi-borylated compounds have received great importance because of their unique synthetic applications [65]. In connection with this, Wu et al. [66] synthesized a series of β-diboryl ketones by utilizing Cu/Pd catalysts (Scheme 50). This protocol tolerates different substituted aryl alkynes; however, this approach is unfavored for internal alkynes. A mono-borylated product was obtained when they used diaryl-substituted internal alkynes. Based on the controlled experiments, the reaction pathway involved in the formation of borocarbonylation of aryl alkynes followed by the hydroboration of the terminal π-system [66].

**Scheme 51:**
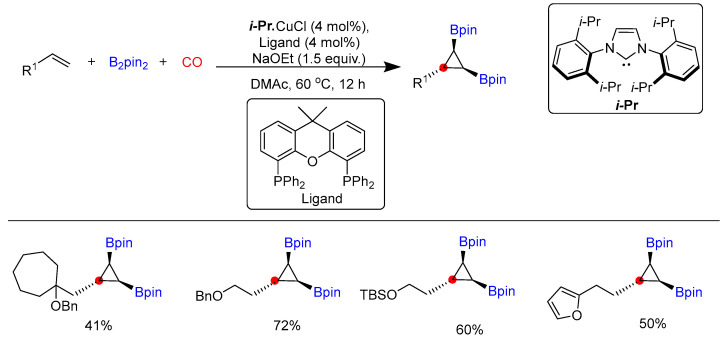
Cu-catalyzed cyclopropanation of alkenes.

Followed by the carbonylative borylation of alkynes, the same research group [67] demonstrated a cyclopropanation of alkenes with diboron and CO catalyzed by copper. This method could generate cyclopropane with two Bpin groups at different sides of the circle with high regioselectivity and up to a 71% yield under the optimized circumstance (Scheme 51).

**Scheme 52:**
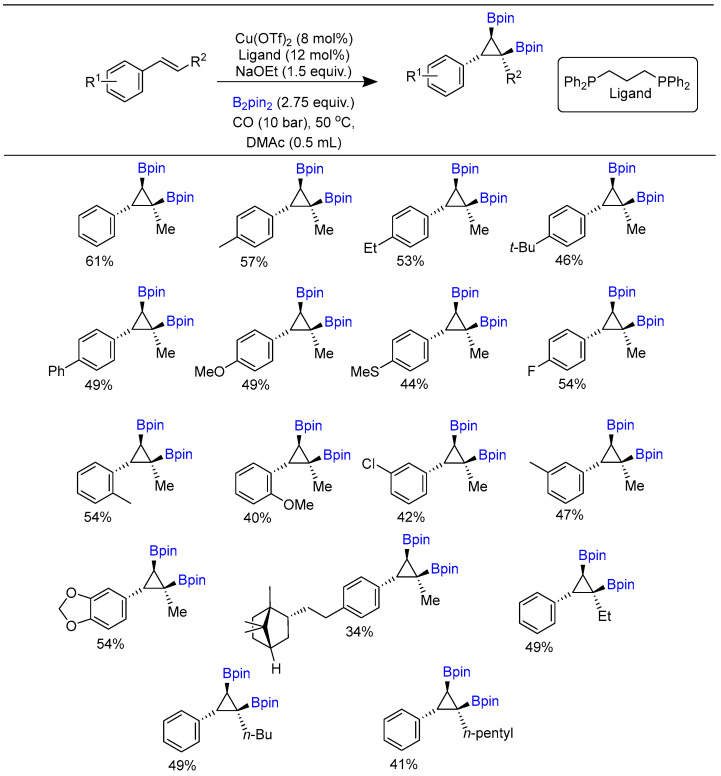
Copper-catalyzed synthesis of cyclopropyl bis(boronates) using different internal aryl alkenes.

Again, the same group [68] explored the preparation of diborylated cyclopropanes from aryl olefins and CO via a copper-catalyzed carbonylation process (Scheme 52). After the identification of the suitable reaction conditions, the authors successfully performed this on both stable internal and terminal aryl olefins. As a consequence, a series of cyclopropyl bis(boronates) were prepared in moderate to good yields with complete diastereoselectivity.

**Scheme 53:**
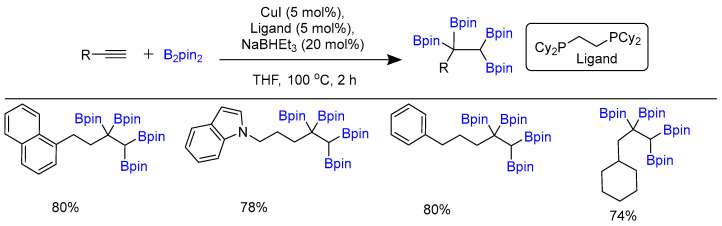
Quadruple borylation of terminal alkynes catalyzed by copper.

Ge et al. [69] successfully achieved a regioselective quadruple borylation of terminal alkynes using CuI and dcpe (1,2-bis(dicyclohexylphosphino)ethane) as a catalytic system (Scheme 53). Under optimal conditions, a wide range of terminal alkynes, including fluorine, silyl, siloxy, sulfide, tertiary amine, amide, and internal alkyne, underwent this reaction smoothly and afforded the expected products in good yields. Of note, alkynes containing heteroaryl groups, such as indole and thiophene, also underwent this reaction to give the desired 1,1,2,2-tetraborylalkanes in 78 and 75% of yields, respectively [69].

**Scheme 54:**
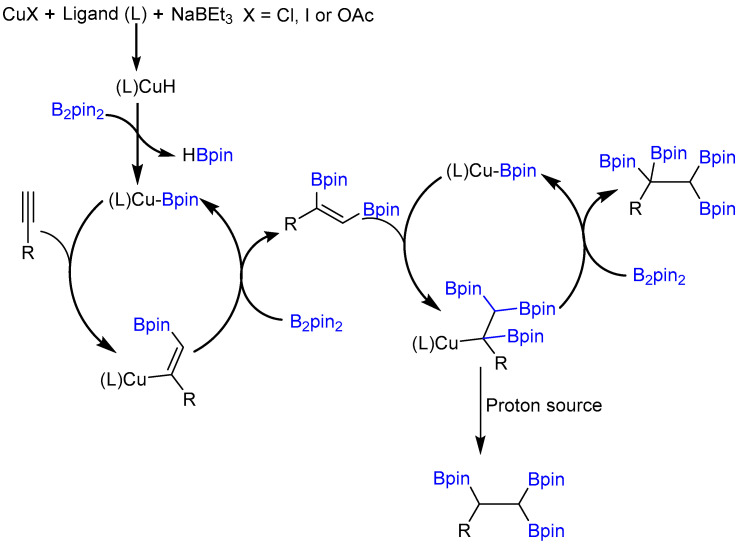
Proposed mechanism for Cu-catalyzed quadruple borylation of alkynes (L = dcpe).

This quadruple borylation reaction was suggested to proceed through the formation of a copper hydride intermediate, copper-boryl species, vinyl copper intermediate, and alkyl copper species. At last, a tetraborylated target product was released with the regeneration of active species as sketched in Scheme 54.

## 6. Conclusions and Outlook

In summary, unquestionably the borylative functionalization of unsaturated molecules has expanded the horizon of copper-catalyzed functionalization. From the initial report of copper-catalyzed borylation, an increasing number of transformations have been reported in the past two decades. The updated progress summarized herein highlights the frontiers and hot directions in the field of Cu-catalyzed borylation chemistry. In the future, the harmonious design of ligand frameworks needs to be focused on achieving regio-, stereo-, and enantio-selective borylation. The advancements in the asymmetric borylation of unactivated alkenes remain challenging; hence, the quest for structurally-novel chiral ligands should be appreciated. In addition, more attention needs to be paid to the Cu-catalyzed borylative alkylation, carbonylative borylation, borylaminomethylation, and aminoboration of unsaturated molecules. In a nutshell, it is envisioned that more creative and efficient multiboration of alkenes and alkynes needs to be undertaken for the construction of more complex-structure molecules. In spite of the advancements, in-depth explorations are required in this exciting area to make it more applicable in the late-stage functionalization of bio-active molecules for drug design. We anticipate that a robust and facile synthetic methodology, that could also find industrial application, needs to be devised in the field of copper-catalyzed borylation reactions. We hope this updated review will be helpful for the field of organic synthesis to develop novel copper-catalyzed borylation reactions.

## Data Availability

Not applicable.
